# Clinical utility of a novel double-band endoscopic band ligation device for consecutive ligation in the management of diverticular bleeding

**DOI:** 10.1016/j.vgie.2025.07.011

**Published:** 2025-08-18

**Authors:** Sayo Daidoji, Yugo Suzuki, Daisuke Kikuchi, Shu Hoteya

**Affiliations:** 1 Department of Gastroenterology, Toranomon Hospital, Tokyo, Japan; 2 Department of Gastroenterology, Toranomon Hospital Kajigaya, Tokyo, Japan

## Abstract

**Background and Aims:**

Endoscopic band ligation is effective for diverticular bleeding; however, conventional devices have drawbacks such as limited visibility, weak suction, and single-use design. Endoligar (Asahi Intecc Co Ltd, Aichi, Japan) addresses these limitations with clear visibility, strong suction, and the ability to perform 2 ligations without removing the endoscope. This report evaluates its clinical utility.

**Methods:**

An 82-year-old man with a history of diverticular bleeding was admitted for hematochezia lasting 3 days. Noncontrast computed tomography and emergency colonoscopy suggested a bleeding diverticulum in the ascending colon. A diverticulum at the hepatic flexure showed signs of a recent hemorrhage, but no active bleeding; a clip was placed for marking. On day 8, a second colonoscopy showed oozing from the previously marked diverticulum, and endoscopic band ligation was performed using the novel device. The first band dislodged, but a second ligation was completed without scope removal.

**Results:**

The procedure lasted 27 minutes, with minimal bleeding and no adverse events. The patient was discharged on day 13.

**Conclusions:**

This is the first clinical case report on this device, to our knowledge. The device enables a second ligation without scope removal, reducing the risk of losing the lesion, and is potentially useful in challenging endoscopic band ligation cases. Further studies are required.

## Introduction

Endoscopic band ligation is an effective treatment for diverticular colonic bleeding.^1^ However, conventional endoscopic band ligation devices have limitations such as restricted visibility, single-use functionality, and inadequate suction power. To address these limitations, a novel device specifically designed for colonic diverticular ligation, the Endoligar (Asahi Intecc Co Ltd, Aichi, Japan), has been developed. It enables up to 2 consecutive ligations without removing the endoscope, offers enhanced visibility, and provides high suction power.^2^

## Case report

**Figure 1:**
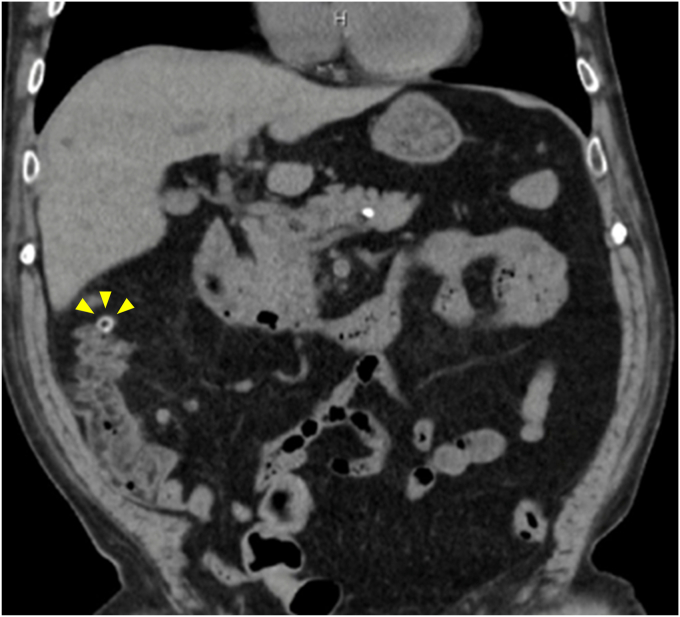
Noncontrast computed tomography at the time of admission. On admission, the patient showed decreased renal function and a history of bronchial asthma, preventing the use of contrast-enhanced computed tomography. Noncontrast computed tomography revealed multiple diverticula in the ascending colon, accompanied by peridiverticular fatty deposits and transudation (*yellow arrowheads*). No significant fluid accumulation is observed in the intestinal tract.

**Figure 2:**
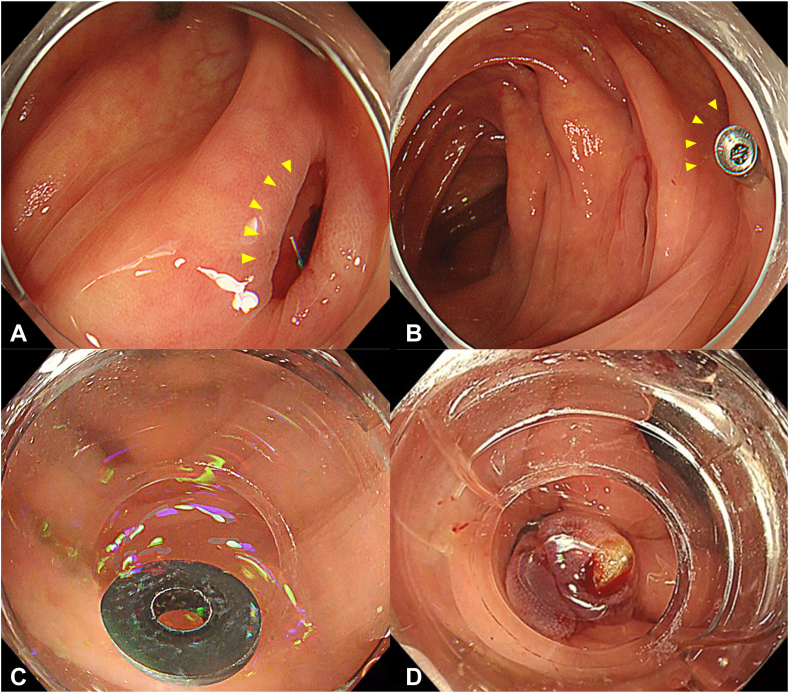
Images of procedure. **A,** Stigmata of a recent hemorrhage of the hepatic fold in the first colonoscopy. A diverticulum with slight traces of blood is observed in the hepatic flexure. Although this could have been an exposed vessel classified as stigmata of a recent hemorrhage, the absence of bleeding on stimulation precluded a definitive diagnosis of stigmata of a recent hemorrhage (*yellow arrowheads*). In addition, spontaneous hemostasis had already been achieved; therefore, no intervention was performed. Instead, a clip is placed near the suspected diverticulum for marking. **B,** Stigmata of the recent hemorrhage of the hepatic fold in the second colonoscopy. During the second colonoscopy, aspiration of the diverticulum suspected to have stigmata of the recent hemorrhage during the first colonoscopy revealed oozing. This suggests that the diverticulum is the source of stigmata of the recent hemorrhage. Consequently, endoscopic band ligation is performed using this device. **C,** Endoscopic view showing the band misfiring during the first endoscopic band ligation. During the second colonoscopy, endoscopic band ligation was performed using this device. However, the first ligation failed when the band misfired. The diverticulum was firm, and the surrounding intestinal wall was edematous, both of which contributed to misfiring. **D,** Endoscopic photograph after the second endoscopic band ligation. The second endoscopic band ligation can be performed immediately by firing the second O-ring without withdrawing the endoscope, and a second ligation was performed immediately using this device. The band was deployed successfully. Elevation of the mucosa, including the diverticulum with stigmata of the recent hemorrhage, was observed after the second endoscopic band ligation procedure.

The patient was an 82-year-old man with a history of 3 previous episodes of diverticular bleeding. He was admitted to our department for evaluation and treatment of hematochezia persisting for 3 days. On the day of admission, noncontrast computed tomography revealed multiple diverticula in the ascending colon, suggesting that the bleeding originated from a diverticular hemorrhage (Fig. 1). Emergency colonoscopy revealed a diverticulum at the hepatic flexure with suspected stigmata of a recent hemorrhage (SRH), specifically a visible vessel; however, no active bleeding was observed (Fig. 2A). Therefore, no active treatment was performed, and only marking was performed near the suspected diverticulum using a clip (Fig. 2B). After he resumed oral intake, the patient experienced multiple episodes of hematochezia. In association with the bleeding, the patient's hemoglobin levels decreased, and his systolic blood pressure dropped to 90 from 100 mm Hg. On day 8 of hospitalization, a second colonoscopy was performed using the PCF-H290I (Olympus, Tokyo, Japan). During this secondary colonoscopy, the suctioning of the previously clipped diverticulum revealed oozing, suggesting suspected SRH, and endoscopic band ligation was performed using this device (Video 1, available online at www.videogie.org). Although the first band was dislodged (Fig. 2C), a second ligation was performed consecutively without removing the endoscope, and the SRH-suspected diverticulum was successfully ligated (Fig. 2D). The procedure time was 27 minutes, and no adverse events were observed. The patient's postoperative course was uneventful, and he was discharged on day 13 after admission.

## Discussion

**Figure 3:**
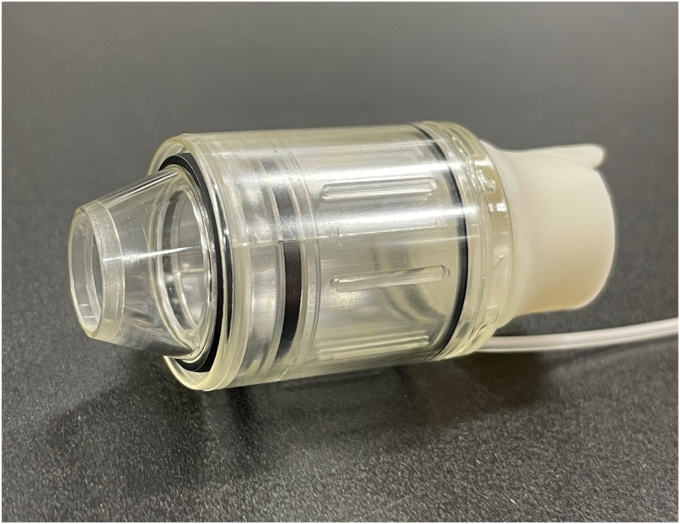
Photograph of the novel device. This novel device is used by attaching it to the distal end of the endoscope.

**Figure 4:**
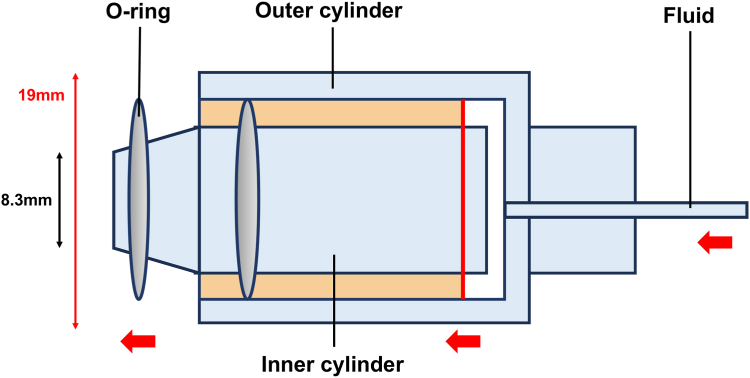
Schematic diagram of the novel device. The 2 O-rings on the outer casing were behind the tip of the endoscope and did not obstruct the endoscopic field of view. After this device was attached to the endoscope tip and the mucosa was drawn into the device lumen by suction, the O-rings were released along the tip taper using air pressure delivered through syringe injection. The first band was released with 2.5 mL of air using a 2.5-mL syringe, and the second band was released with 5.0 mL of air using a 5.0-mL syringe.

A novel endoscopic band ligation device was recently approved, and reports of real-world clinical outcomes remain limited. To our knowledge, this is the first case report of endoscopic band ligation with this device (Fig. 3). A large retrospective cohort study on diverticular bleeding revealed that identifying a suspected SRH and performing endoscopic treatment are beneficial, particularly in the right side of the colon, where endoscopic therapy is superior to conservative treatment.^3^ Furthermore, among endoscopic treatments, endoscopic band ligation is more effective than clipping in the right side of the colon.^4^ However, in clinical practice, endoscopic band ligation failure occurs in certain cases. For example, large diverticular openings, multiple diverticula, and edematous changes, as observed in this case, increase the likelihood of endoscopic band ligation deployment failure.^1^ These conditions may lead to insufficient suction and reduced band fixation, thereby increasing the likelihood of treatment failure. In such situations, this novel device is expected to provide significant clinical benefits. The primary feature of this device is the presence of 2 preloaded O-rings, which allow for a second ligation without removing the endoscope (Fig. 4). This feature reduces the risk of losing sight of the lesion and enables consecutive ligations, even if the first band ligation fails, as demonstrated in this case. In addition, the O-rings are preloaded within the outer tube to minimize the obstruction of the visual field and potentially facilitate the identification of the bleeding point during reinsertion. Furthermore, the tapered tip, with an outer diameter of 8.3 mm, provides strong suction capability and may contribute to improved procedural success rates. This effect has also been confirmed in preclinical animal studies.^2^ However, this device has certain limitations; the maximum outer diameter of 19 mm may make insertion difficult in certain cases, such as in those with stenosis or severe flexure of the sigmoid colon.^4^ Table 1 summarizes the differences between devices based on structural characteristics. This case demonstrates that this device was effective in achieving hemostasis in a patient with recurrent diverticular bleeding. This novel device is effective in cases where endoscopic band ligation is challenging, particularly in patients with conditions associated with endoscopic band ligation deployment failure. Future prospective multicenter randomized controlled trials are warranted to confirm the effectiveness of this treatment.

**Table 1:** Comparison between this device and other band ligation devices

Generic name	Trade name	Intended use	Scope compatibility (diameter, mm)	Distal hood diameter (mm)	Length (mm)	Number of O-rings	Hood color/hood structure
Endoscopic band ligation device	Endoligar (Asahi Intecc Co Ltd, Aichi, Japan)	Colonic diverticular bleeding, internal hemorrhoids	Colonoscope (11.5)	18.5	30	2 (dual-shot)	Transparent Tapered External O-ring mount
Endoscopic esophageal varix ligation device	Speedband Superview Super7 (Boston Scientific Corporation, Marlborough, Mass, USA)	Esophageal varices	Upper endoscopes (9.0-11.0)	30	30	Up to 7	Transparent Nontapered Large Internal O-ring mount
Endoscopic esophageal varix ligation device (conventional)	Pneumoactivated esophageal variceal ligation device (MD-48710U; Sumitomo Bakelite Co, Ltd, Tokyo, Japan)	Esophageal varices	Upper endoscopes (11-12)	12-14	30	1	Semitransparent Nontapered Short hood Internal O-ring mount
Endoscopic band ligation device (modified)	Endoscopic band ligation device (MD-48910B, MD-48912B, MD-48913B; SB-Kawasumi Co, Ltd, Tokyo, Japan)	Colonic diverticular bleeding, internal hemorrhoids, rectal NET	Colonoscopes (10.5-13.8)	14	30-32	1	Transparent Nontapered Slim profile Internal O-ring mount

*NET*, Neuroendocrine tumor.

## Patient consent

Written informed consent was obtained from the patient for the publication of this case report and the accompanying images.

## Disclosure

All authors disclosed no financial relationships.

## Supplementary data

Video 1 Successful endoscopic band ligation with this device for diverticular bleeding in the second colonoscopy.

R1 Legends for video and figures
