# Supplementary material for: Clinical utility of a novel double-band endoscopic band ligation device for consecutive ligation in the management of diverticular bleeding
Source: VideoGIE. 2025 Aug 18;11(1):21–4. doi: 10.1016/j.vgie.2025.07.011 (PMC12827021; doi:10.1016/j.vgie.2025.07.011)
Supplement: R1 Legends for video and figures [file mmc2.docx]

**Figure legends**

**Figure 1: Non-contrast computed tomography at the time of admission**

Upon admission, the patient showed decreased renal function and a history of bronchial asthma, preventing the use of contrast-enhanced computed tomography. Non-contrast computed tomography revealed multiple diverticula in the ascending colon, accompanied by peridiverticular fatty deposits and transudation (yellow arrowheads). No significant fluid accumulation is observed in the intestinal tract.

**Figure 2: Images of procedure**

**Figure 2a: Stigmata of recent hemorrhage of the hepatic fold in the first colonoscopy**

A diverticulum with slight traces of blood is observed in the hepatic flexure. Although this could have been an exposed vessel classified as stigmata of recent hemorrhage, the absence of bleeding upon stimulation precluded a definitive diagnosis of stigmata of recent hemorrhage (yellow arrowheads). Additionally, spontaneous hemostasis had already been achieved; therefore, no intervention was performed. Instead, a clip is placed near the suspected diverticulum for marking.

**Figure 2b: Stigmata of recent hemorrhage of the hepatic fold in the second colonoscopy**

During the second colonoscopy, aspiration of the diverticulum suspected to have stigmata of recent hemorrhage during the first colonoscopy revealed oozing. This suggests that the diverticulum is the source of stigmata of recent hemorrhage. Consequently, endoscopic band ligation is performed using this device.

**Figure 2c: Endoscopic view showing the band misfiring during the first endoscopic band ligation**

During the second colonoscopy, endoscopic band ligation was performed using this device. However, the first ligation failed when the band misfired. The diverticulum was firm and the surrounding intestinal wall was edematous, both of which contributed to misfiring.

**Figure 2d: Endoscopic photograph after the second endoscopic band ligation**

The second endoscopic band ligation can be performed immediately by firing the second O-ring without withdrawing the endoscope, and a second ligation was performed immediately using this device. The band was deployed successfully. Elevation of the mucosa, including the diverticulum with stigmata of recent hemorrhage, was observed after the second endoscopic band ligation procedure.

**Figure 3: Appearance of the novel device**

This novel device is utilized by attaching it to the distal end of the endoscope.

**Figure 4: Schematic diagram of the novel device**

The two O-rings on the outer casing were behind the tip of the endoscope and did not obstruct the endoscopic field of view. After this device was attached to the endoscope tip and the mucosa was drawn into the device lumen by suction, the O-rings were released along the tip taper using air pressure delivered through syringe injection. The first band was released with 2.5 mL of air using a 2.5-mL syringe, and the second band was released with 5.0 mL of air using a 5.0-mL syringe.

**Video legend**

**Video 1: Successful endoscopic band ligation with this device for diverticular bleeding in the second colonoscopy**
